# The Relevance of Diet, Physical Activity, Exercise, and Persuasive Technology in the Prevention and Treatment of Sarcopenic Obesity in Older Adults

**DOI:** 10.3389/fnut.2021.661449

**Published:** 2021-05-24

**Authors:** Josje D. Schoufour, Michael Tieland, Rocco Barazzoni, Somaya Ben Allouch, Joey van der Bie, Yves Boirie, Alfonso J. Cruz-Jentoft, Doris Eglseer, Eva Topinková, Bart Visser, Trudy Voortman, Amalia Tsagari, Peter J. M. Weijs

**Affiliations:** ^1^Faculty of Sports and Nutrition, Centre of Expertise Urban Vitality, Amsterdam University of Applied Sciences, Amsterdam, Netherlands; ^2^Department of Medical, Surgical and Health Sciences, University of Trieste, Trieste, Italy; ^3^Digital Life Research Group, Faculty of Digital Media and Creative Industry, Amsterdam University of Applied Sciences, Amsterdam, Netherlands; ^4^University Clermont Auvergne, Human Nutrition Unit, INRA, CRNH Auvergne, CHU Clermont-Ferrand, Clinical Nutrition Department, Clermont-Ferrand, France; ^5^Servicio de Geriatría, Hospital Universitario Ramón y Cajal (IRYCIS), Madrid, Spain; ^6^Department of Nursing Science, Medical University Graz, Graz, Austria; ^7^First Faculty of Medicine, Department of Geriatrics, Charles University, Prague, Czechia; ^8^Faculty of Health and Social Sciences, University of South Bohemia, Ceske Budejovice, Czechia; ^9^Faculty of Health, Center of Expertise Urban Vitality, Amsterdam University of Applied Sciences, Amsterdam, Netherlands; ^10^Department of Epidemiology, Erasmus MC University Medical Center Rotterdam, Rotterdam, Netherlands; ^11^Department of Clinical Nutrition, KAT General Hospital, Athens, Greece; ^12^Department of Nutrition and Dietetics, Amsterdam University Medical Centers, Amsterdam Public Health Institute, VU University, Amsterdam, Netherlands

**Keywords:** sarcopenic obesity, nutrition, physical activity, blended care, eHealth, elderly

## Abstract

The aging population faces two conditions that threaten healthy aging: high fat mass (obesity) and low muscle mass and function (sarcopenia). The combination of both—referred to as sarcopenic obesity—synergistically increases the risk of adverse health outcomes. The two conditions often co-occur because they reinforce each other and share common etiologies, including poor nutrition and inactivity. All aging people are at risk of gaining weight and losing muscle mass and could benefit from improvements in physical activity, exercise and dietary intake. one specific window of opportunity is during the transient time of retirement, as older adults already need to restructure their daily activities. It is key to change lifestyle behavior in a sustainable manner, providing scientifically proven, personalized, and acceptable principles that can be integrated in daily life. Health technologies (e.g., applications) can provide promising tools to deliver personalized and appealing lifestyle interventions to a large group of people while keeping health care costs low. Several studies show that health technologies have a strong positive effect on physical activity, exercise and dietary intake. Specifically, health technology is increasingly applied to older people, although strong evidence for long term effects in changing lifestyle behavior is generally lacking. Concluding, technology could play an important role in the highly warranted prevention of sarcopenic obesity in older adults. Although health technology seems to be a promising tool to stimulate changes in physical activity, exercise and dietary intake, studies on long lasting effects and specifically targeted on older people around the time of retirement are warranted.

## Introduction

Two severe public health threats strike Europe: obesity and sarcopenia, defined as loss of skeletal muscle mass and function (1, 2). Both obesity and sarcopenia predispose for comorbidity, immobility, dependency, disability, low quality of life, and many unhealthy life years (3–5). Obesity and sarcopenia synergistically reinforce each other in vicious cycles of increases in fat mass and muscle loss through reduced mobility, dependency and disability (2). The concept of sarcopenic obesity has been introduced to describe the association and co-occurrence of these two phenotypes (3, 6). The exact prevalence of sarcopenic obesity highly depends on the criteria used and the awareness of this relatively new condition, as obesity can mask the reduction in muscle mass (6), but is estimated around 12.6% in men and 33.5% in women by the National Health and Nutrition Examination Survey. These rates highly increase with age, reaching 48% in men and 27,5% in women, in those aged 80 years and above (4, 7). The negative clinical impact of sarcopenic obesity on health, quality of life and health cots is enormous (8–10). This negative impact is particularly present in the aging population, where physical inactivity, limited exercise and poor nutritional intake (both over and under consumption of nutrients) may even further lead to metabolic and clinical complications (11–13). To improve physical activity, exercise and nutrition, digital technologies have caught the interest of behavioral scientists and technology developers (14). The use of personalized digital health technologies (eHealth) and blended care (combination of eHealth and face-to-face contact) can be a promising strategy to support people in their challenge to follow a healthier lifestyle (15, 16). Digital health technologies can be used to target the upcoming population of older people, a population that is expected to increasingly use internet and mobile devices such as smartphones and tablets. Possible, one specific window of opportunity is during the transient time of retirement, as older adults already need to restructure their daily activities. In this paper we describe how and why older adults, specifically those in the phase of retirement, could benefit from health technologies focused on dietary intake, physical activity and exercise. To understand the need for such interventions we provide a brief overview of the interplay between sarcopenia and obesity at old age and the essence to focus on both dietary intake, physical activity and exercise.

## The Interplay Between Obesity and Sarcopenia in Old Age

Aging *per se* leads to gradual changes in body composition with increases in fat mass and decreases in muscle mass. Aging is associated with a reduction in skeletal muscle fiber size and number, a reduction in muscle mass and quality, a reduction in oxidative capacity and with an increased muscle fat infiltration. All together leading to a reduction of muscle strength and power, subsequently causing mobility disability and functional impairment, a condition named sarcopenia (2). Muscle mass generally peaks at the end of the third decade of life, after which it declines by approximately 0.37 and 0.47% per year in women and men, respectively. The etiology of this age-related muscle loss is multifactorial (2). It may result from metabolic conditions including chronic inflammation, hormonal changes, oxidative stress, skeletal muscle mitochondrial and stem cell dysfunction (17–20) as well as lifestyle factors including sedentary behavior, low physical activity and poor nutrition including protein and micronutrient deficiencies (21, 22). Aging is also associated with relative or absolute increments in body fat, which may lead to or worsen pre-existing overweight and obesity (23–25).

Furthermore, old age epitomizes the synergistic interactions between obesity and sarcopenia. A reduction in muscle mass directly leads to lower whole-body metabolic rate and reduced oxidative capacities. This leads to lower energy expenditure from basal metabolism, which may contribute to further weight gain, which is mostly fat mass, particularly if protein intake is too low (26). In turn, excess body fat may enhance age-associated systemic and tissue inflammation, oxidative stress, mitochondrial dysfunction and insulin resistance with direct negative impact on skeletal muscle (3, 27, 28). For example, in the presence of positive energy balance and limited adipose tissue expandability to store excess lipids, ectopic muscle fat infiltration commonly occurs. This muscle fat infiltration is associated with poor muscle quality (3, 29) and is associated with insulin resistance (30) leading to further reduction of muscle strength and endurance (31, 32). Furthermore, both obesity and low muscle mass may be associated with anabolic resistance—the limited skeletal muscle protein synthetic response after an anabolic stimulus, for example, protein intake or physical activity (33–38). Nevertheless, how this mechanism works remains to be elucidated as recent studies did not find an association of obesity or fat intake on muscle protein synthesis (39, 40).

Other, age-associated, causes that have been suggested are among others lipotoxicity, inflammation, insulin resistance (type II diabetes) and low physical inactivity (41).

On top of the above-mentioned metabolic interactions, sarcopenia and obesity also interact in daily life. Obesity often promotes inactivity, as routine activities of daily living—walking, climbing stairs, maneuvering in public spaces—are more difficult for obese individuals, let alone participation in leisure activities or exercise programs (42). In addition, a frequently forgotten issue is that muscles in obese persons need to move a higher body mass, so even an apparently normal muscle strength may not be enough to perform activities that would be feasible with a lower body weight. Furthermore, another important cause of sarcopenic obesity may stem from weight cycling that can occur in obese people. Indeed, it has been shown that successive periods of weight loss and regain promote sarcopenia through a higher recovery of fat mass than fat free mass during weight regain (43, 44). Specifically, weight loss alone is already a risk for sarcopenia as approximately 25% of the lost weight is attributable to fat free mass (26). Through all of the above combined mechanisms, obesity leads to sarcopenia and vice versa with strong synergistic vicious cycles that are particularly dangerous in older adults (3, 4, 12). Older adults should therefore be stimulated to prevent weight gain and preserve muscle mass.

## Lose the Fat, Preserve the Muscle—the Essence of Diet, Exercise and Physical Activity

To prevent or treat sarcopenic obesity, it is essential to prevent weight gain or lose fat mass, while preserving muscle mass (45–49). Exercise is a promising tool to tackle both sarcopenia and obesity. First of all because exercise can cause, if not compensated with higher caloric intake, a negative energy balance leading to loss of fat mass (50, 51). However, people tend to overcompensate increased physical activity by reducing other activities (more sitting versus walking) and/or increasing their energy intake (52). Second, exercise and physical activity can have beneficial effects on physical performance (e.g., strength, gait speed, balance) and stimulates muscle mass gain or maintenance (53). Recent guidelines from the WHO state that older adults should undertake regular physical activity of at least 150–300 min of moderate-intensity aerobic physical activity or 75–150 min of vigorous-intensity aerobic physical activity for substantial health benefits. In addition, older adults should do muscle strengthening activities at moderate or greater intensity that involve all major muscle groups twice weekly. General guidelines for a resistance-type exercise training suggest to train with a load of 60–80% of 1RM, 3–4 sets per muscle group with 1–2 min rest intervals, allowing muscle mass preservation and/or gain in older adults (54, 55). These guidelines, however, are mainly based on apparently healthy (older) adults and certainly more research is needed in more frail or sarcopenic obese adults. Studies that do include sarcopenic obese older adults generally observe beneficial changes in body composition and physical performance following resistance-type exercise training (55–59).

In addition to exercise, nutrition is key to counteract both sarcopenia and obesity. In essence, obesity is a result of an excess consumption of energy as compared to individual needs, whereas sarcopenia can be caused by an inadequate dietary intake, particularly protein. A hypocaloric diet may therefore be very efficient to lose weight, but with insufficient proteins, it can be detrimental for muscle mass maintenance (48, 55). In order to optimally promote muscle protein synthesis and to maintain or to regain muscle mass, it has been recommended to reach a dietary protein intake of 1.0–1.2 g/kg body weight instead of the recommended 0.8 g/kg body weight in healthy adults (60). During weight loss a higher than 1.2 g/kg body weight level is even recommended (61). In addition to the amount of protein, other aspects of an overall healthy diet play an important role in promoting muscle health and body composition including vitamins, antioxidants, protein quality and timing of protein, and the overall quality of the diet (21, 62–65).

To target both sarcopenia and obesity in sarcopenic obese older adults, a combination of both exercise and nutrition seems most appropriate. Energy restriction alone can be successful for body weight loss, but can be costly is terms of losses in muscle mass (48). Indeed, weight loss studies show the ability to improve physical performance to some extent with exercise or diet alone, but are more effective when the exercise and diet are combined (66). The benefits of protein supplementation during prolonged exercise training in both younger and older subjects has been confirmed by meta-analysis (67). Concluding, current evidence suggests that a modest hypocaloric diet with sufficient proteins combined with exercise can be effective to counteract sarcopenic obesity in older adults. Nevertheless, feasible studies that combine nutrition and exercise strategies and take into account feasibility are highly warranted in specifically older people with sarcopenic obesity.

## Window of Opportunity: Retirement

It is important to adapt a healthy lifestyle early in the lifespan to prevent overweight, obesity and sarcopenic obesity in later life (68). Even so, interventions to improve lifestyle in later life can still be of paramount importance. For several reasons we believe that one specific window of opportunity to improve lifestyle behavior is during the transient time of retirement. First, changing a lifestyle is by no means easy. Older adults that go through the work-retirement transition already need to restructure their lifestyle, habits and daily activities. Second, generally retirees are motivated to establish new daily routines, to enhance opportunities for social interactions and personal challenges, and to increase recreational physical activity to satisfy needs that had been previously fulfilled by their work (69). Third, with current life expectancy, adopting a healthy lifestyle around the age of retirement could offer sufficient time to prevent unhealthy aging and dependency in later life for a substantial period of time. Last, demographic changes make that there is an upcoming wave of people that will enter retirement. Effective interventions in this large group of people can therefore have major public health impact.

Nevertheless, despite the motivation and timing, making actual changes to improve lifestyle behaviors proofs to be difficult. Indeed, unfortunately, during the transition from working life to retirement, the risk to lose muscle mass and gain fat mass may increase rapidly (70–73). People may spend more leisure time with feasting food habits while leading an inactive lifestyle. This leads to an acceleration in the ongoing process of age-related gain in fat mass and loss of muscle mass, causing a high risk of developing sarcopenic obesity. Although a large proportion of older adults experience a reduction in physical activity and gain weight during the transition from work to retirement, there are important differences between subgroups (74, 75). For example, it was shown that men retiring from strenuous jobs tend to gain weight, while those retiring from sedentary jobs usually experience no weight gain (70, 71), and persons who are already overweight often gain more weight in the retirement phase than persons who start retirement with normal weight (72). Also, older retirement age, higher occupational status and fewer chronic diseases were associated with increases in physical activity level in retirement (76). In order to stimulate a more healthy lifestyle after retirement there is a need for effective intervention strategies that consider individual as well as population-based and environmental factors and specifically stimulate sustainable behavioral changes. In previous lifestyle interventions that were successful on the short term, dietary habits and physical activity levels often revert to the levels before the intervention period (77, 78). Interventions aiming to increasing physical activity among people in retirement emphasize the importance of provide tailored and adaptable exercise programs (79, 80). Digital technologies can help to stimulate long term changes, take into account personal motives and threats, previous occupation, goals, opportunities and wishes, account for population and environmental factors, and potentially reaches a large number of individuals at relatively low costs (81, 82). Upcoming older adults and retirees have less digital literacy than their older peers, making them more qualified to use digital technology (83).

## Innovative Strategies

Digital technologies are promising to stimulate sustainable health behavioral change. Web-applications, wearable devices and mobile health applications, for example, may comprise tailored information, behavior change techniques including goalsetting and monitoring which motivate users to change behavior and manage their disease adequately at relatively low costs (81, 82). Since technologies play a key role in everyday life, there is a major increase in the development and use of mobile lifestyle interventions. It is expected that the face-to-face management of health disorders will gradually shift to a more technology supported approach, reducing the large burden on health personnel and resources (84). Successful health technologies are frequently based on well-grounded cognitive behavioral strategies incorporating psychological elements. Persuasive technology aims to change people's attitudes and behaviors and is an essential instrument to enhance online and offline adherence (85, 86), promote self-management abilities (87, 88) and feeling of competence (89). Persuasive technology for supporting health behavior of older adults often applies techniques such as: personalisation, suggestion, goal-setting, simulation and reminders (59, 90–92). However, it is not always clear how these theories lead to design criteria in health technology and the mechanisms of effective intervention studies are understudied (93, 94).

Previous reviews and meta-analyses have shown that digital health technologies can be an effective vehicle to promote a healthy lifestyle among people of various ages (81, 95–99). Tailored technology-supported physical activity, exercise and nutrition interventions have demonstrated positive effects on physical activity levels, dietary fat intake, fruit and vegetable consumption (89, 100, 101), body weight, blood pressure, fasting plasma glucose and blood lipids (102, 103). Also in older adults aged 55 years and over, digital technology has shown beneficial effects on lifestyle behavior, although the long-term effects were less clear (96, 97). Specifically, there are several studies that show effectiveness of digital health technologies on increasing protein intake among community dwelling elderly (104, 105). Moreover, home-based digital technology-based programs showed successful results in improving multiple facets of sarcopenia including muscle mass and muscle strength in older adults (99, 106, 107). The use of blended care, the combination of face-to-face counseling with health technology, is more and more used (108, 109). These blended interventions are more effective than “stand-alone” interventions without professional guidance (110). The integration of online and offline leads to improved adherence rates which is crucial for the effectiveness of digital health technologies (82). The personal contact that offers among others empathy, personal assistance and coaching, and warmth could be of specific importance for older adults (94). One good example of a blended care intervention for older adults, that was built on well-described theoretical principles of behavior change is the VITAMIN trial (111). This trial was developed to counteract the decline in physical functioning and sarcopenia using a blended-care home-based exercise and dietary protein intervention and showed positive effects on among others muscle mass, muscle strength and protein intake, but not on physical performance in healthy older adults (55 years and over) (110, 112). However, whether this approach also works for frail older people and people with or at risk for sarcopenic obesity should be further explored.

Designing and implementing digital technology for older adults demands additional caution. When implementing persuasive technology for vulnerable people, care needs to be taken for ethical concerns. For example taking into account users' interests and their autonomy (113). Furthermore, when designing technology for long term adherence by older adults, personal influential factors as digital competency and attitude toward digital solutions need to be taken into consideration (89). Although the majority of older adults have a positive attitude toward eHealth, not all older adults own a smartphone or are able to the device in its full potential (114). With the rise of adoption rate of smartphones among older adults, the non-adoption group might become smaller over the years (83). Still, at this moment it remains highly relevant, as there are many countries where large portions of older adults not frequently use modern technology as smartphones and social media (115). Furthermore, visual impairments and reduced fine motor skills become more present as the age rises. These personal age-related factors are not fully taken into consideration in current applications (114). Even so, when eHealth is used in a proper, individualized and, safe way, older adults seem to have a positive attitude toward digital technology (116), which offers opportunities to develop eHealth applications to improve physical activity, exercise and dietary intake in a sustainable manner (82). However, there is a need for well-designed health applications for older people in general, or those going through retirement, that aim to prevent or treat sarcopenic obesity.

## Conclusion

There is an urgent need to prevent and treat sarcopenic obesity. To achieve this, a combination of limited energy intake with adequate dietary protein and sufficient exercise and physical activity are needed (45–49, 55, 117). Sole focus on physical activity during weight loss may lead to insufficient effects regarding both the reduction of fat mass and the preservation of muscle mass. Sole focus on energy restriction can be successful for body weight loss, but can be costly is terms of losses in muscle mass (48). With the rapid development of technology that is getting more accessible and easier to use for older adults, there is a huge potential to develop health technologies that target the growing population of 50 years and above. More specifically, health technology can be applied to the growing group of people that enter retirement and help them to build long-term changes. This group is at high risk for sarcopenic obesity, but motivated to adapt a healthy lifestyle behavior in their new daily routines. For optimal results these innovative strategies have to be based on behavior change principles and are preferably applied as blended care.

## Data Availability Statement

The original contributions presented in the study are included in the article/supplementary material, further inquiries can be directed to the corresponding author/s.

## Author Contributions

JS, MT, RB, SB, JB, YB, AC-J, DE, ET, BV, TV, AT, and PW contributed to conception of the paper. JS wrote the first draft of the manuscript. MT, RB, SB, JB, YB, AC-J, DE, ET, BV, TV, AT, and PW wrote sections of the manuscript and contributed to manuscript revision, read, and approved the submitted version. All authors contributed to the article and approved the submitted version.

## Conflict of Interest

The authors declare that the research was conducted in the absence of any commercial or financial relationships that could be construed as a potential conflict of interest.
